# Admitting privileges: A construction ecology perspective on the unintended consequences of medical school admissions

**DOI:** 10.1007/s10459-023-10210-5

**Published:** 2023-03-01

**Authors:** Janelle S. Taylor, Claire L. Wendland, Kulamakan (Mahan) Kulasegaram, Frederic W. Hafferty

**Affiliations:** 1 Department of Anthropology, University of Toronto, Toronto, ON Canada; 2 Department of Anthropology, Department of Obstetrics & Gynecology, University of Wisconsin-Madison, 5436 Sewell Social Sciences Building 1180 Observatory Drive, 53708 Madison, WI USA; 3 Department of Family and Community Medicine, Wilson Centre for Research in Education, University of Toronto, Toronto, ON Canada; 4 Division of General Internal Medicine and Program in Professionalism and Values, Mayo Clinic, Rochester, MN USA

**Keywords:** Admissions, Shadowing, Global health experiences, Medical scribes, Premedical students, Privilege, Social inequality, Construction ecology

## Abstract

Medical-school applicants learn from many sources that they must stand out to fit in. Many construct self-presentations intended to appeal to medical-school admissions committees from the raw materials of work and volunteer experiences, in order to demonstrate that they will succeed in a demanding profession to which access is tightly controlled. Borrowing from the field of architecture the lens of construction ecology, which considers buildings in relation to the global effects of the resources required for their construction, we reframe medical-school admissions as a social phenomenon that has far-reaching harmful unintended consequences, not just for medicine but for the broader world. Illustrating with discussion of three common pathways to experiences that applicants widely believe will help them gain admission, we describe how the construction ecology of medical school admissions can recast privilege as merit, reinforce colonizing narratives, and lead to exploitation of people who are already disadvantaged.

## Reframing admissions as a social reality

Within the medical education literature, “medical-school admissions” is generally understood as a phenomenon defined by the work that admissions committees do, especially the process of selecting individual applicants from the pool. Questions asked and answered concern how that work is done, how it could be done better, and how different ways of doing it have consequences for individual applicants, for medical schools, and for medicine as a profession (Hecker & Norman, 2017). Implicit in such discussions is an image of admissions as a fundamental problem of hydraulics: a vast flood of applicants approaches a narrow sluice gate, and the question generally is how best to adjust this gate to make sure that it admits exactly the right quality and mixture as well as volume.

We take a different view. Our interest in admissions encompasses not only its effects within medicine, downstream from the sluice gate, but also the broader social landscape upstream that has generated this flood of applicants. Where have they come from? And how have they been channeled in this direction–at what costs, and to whom?

We propose that medical-school admissions should be considered as part of an ecology that encompasses far more than just the work of admissions committees. It includes all the social processes entailed in the production of the applicant pool, many of which lie outside the direct view and beyond the control of admissions committees. Admissions, in this perspective, includes not only applicants who are admitted, but applicants who never gain admission, as well as others who never made it to the stage of applying to become a physician. It also includes the many people who advise pre-medical students (often for a price) on how to gain admission – regardless of whether the medical profession has authorized them to do so, or whether their advice is good. The broader ecology of medical-school admissions has consequences for people and communities affected by the activities that would-be physicians, following such advice, pursue on their way to the applicant pool. This reframing of admission from a proximal problem of engineering hydraulics to a social reality with proximal and distal effects constitutes a theoretical intervention because it brings into view new dimensions of the phenomenon, raises new questions, and makes possible new insights.

Discussions of sustainability in architecture offer a conceptual reframing that we borrow as a theoretical lens. Sustainability, many voices in the field of architecture now insist, requires not only that one design a building to be energy efficient, but also that one take a comprehensive view of the impacts of the resources required for its construction (AIA, 2019). In this perspective, the building that is the final product figures only as a relatively small node in a vast, dispersed “construction ecology” that also encompasses the distant sources of the materials used to build it, the global shipping systems along which those materials move, and the human labor and energy required to create them all and put them into place. As architect Kiel Moe puts it, taking this perspective “further helps understand what all building is and does, and helps us architect in different ways accordingly” (Moe, 2021: 30).

It is in the spirit of urging a similarly broad view of medical-school admissions that we turn now to consider the construction ecology of medical-school admissions. Reframing admissions in construction-ecology terms moves us from a sluice gate model of admissions to a model that considers the entire supply chain of applicants. It is particularly useful in understanding barriers to building the diverse, equitable and inclusive medical profession that is needed for the 21st century. To look carefully at the ecology of admissions directs attention toward a broader, systems perspective on equity. It is well understood already that individual applicants bring very unequal forms and degrees of what the sociologist Bourdieu (1987) called “cultural capital,” i.e. the implicit and embodied forms of knowledge (including tastes in music or art, and styles of speech or dress) that both convey and confer advantage in a highly stratified social order. Our point, however, is different: admissions as a social reality has iatrogenic effects that ramify far beyond medical schools, and beyond applicants themselves, into the broader social and cultural world.

Designing a better profession requires looking at those distal effects, much as designing a more sustainable building requires looking beyond the materials available at the nearest big-box store. Construction supply chains are complex and multinational. In search for competitive advantage, suppliers are often reluctant to be transparent about sourcing and labor practices. Architects and planners then have “ample opportunity to look away from issues that are too remote—or too embedded—to tackle” (Crates, 2016:6). A construction-ecology perspective can help architects not to look away, but rather to increase value, prevent waste and exploitation, and plan for an uncertain future. We believe that understanding the ecology of medical-school admissions has similar potential.

## Building plans

Medical-school applications are not just intended to confirm that applicants have the academic skills and preparation needed to survive medical training. Taken as a whole, applications are also supposed to reveal the personal qualities desired in a physician (altruism, empathy, grit, etc.); demonstrate that the applicant knows something about what medicine really is, and is committed to it; and—in recent years—provide information about challenges overcome, so that admissions committees can take “distance traveled” into account as they attempt to diversify entering classes (AAMC, 2022b). Standardized or semi-standardized assessments and grades alone cannot accomplish any of these important goals. In addition to transcripts and exam scores, therefore, medical schools ask applicants for a history of work activities and volunteer experiences, and for reflections on which of these activities was most valuable (AAMC, 2022a). Applicants must also provide essays that convert their experiences into narratives; as the American Association of Medical Colleges (AAMC) notes, these essays are “your opportunity to distinguish yourself from other applicants” (AAMC, 2022b).

Many informal and formal sources guide would-be doctors on how to seek raw materials for these essays: experiences that will enable them to construct narratives that present desirable kinds of selves. Unofficial advice circulates through the pre-medical rumor mill. Official sources, such as websites of medical schools or application services (e.g., American Medical College Application Service) and pre-medical advisors at universities and colleges, steer students toward the kinds of activities expected and offer guidance for strategically presenting these experiences to admissions committees. In addition, a huge commercial industry markets advice books, prep courses, and even personal application coaches to those who can pay. These sources describe ways of telling stories that will depict student applicants as “unique” but at the same time show them to fit an imagined physician archetype (White et al., 2011; 2012). This guidance is evident in a broad sample of advice-industry materials (see e.g., Dowhan et al., 2014; the Crimson Brand Studios 2019). One guidebook, for instance, urges students to convey in their essays “the type of person [reviewers] would trust in a life or death situation” (Dowhan et al., 2014:13).

Eager consumers in this marketplace of advice, medical-school applicants work hard to create and present to admissions committees a certain kind of self: experienced, committed, eager, knowledgeable, and altruistic. Their applications incorporate a variety of activities that testify to these unique-yet-archetypal selves, described more directly in personal-statement essays and during interviews. Studying this process, researchers in Canada found that “applicants knew what ‘proper’ answers would be and modulated their own to fit, understanding that they needed to ‘sell yourself’ to stand out” (White et al., 2012:6).

Applicants’ experiences and the stories they tell about them do matter to many admissions committees. (One of us recalls, for example, sweeping applicant files that lacked evidence of meaningful exposure to primary care into a large “no” pile.) There is, however, little research to support claims that extracurricular activities predict success in medical school. Similarly, personal essays have not been shown to predict clerkship performance (Dong et al., 2013; Kreiter & Axelson, 2013). In fact, there is no consensus on how to evaluate such essays (Kulatunga-Moruzi & Norman, 2002). Whether these accumulated self-narratives actually help admissions committees select the applicants best suited to succeed and embody the values of the profession remains an open question—one that we are not going to answer (Siu & Reiter, 2009).

## What admissions is and does: Harmful effects in admissions’ construction ecology

Our purpose, instead, is to draw attention to broader damage created by the medical-school application process. Whatever they do or do not achieve within medical schools, we argue that would-be doctors’ efforts to create compelling applications and demonstrate that they are made of “the right stuff” for medicine come at a cost— to them, but also to society.

We four co-authors have witnessed some of these unintended consequences. Two of us have taught thousands of pre-medical hopefuls. Two of us have been members of medical-school admissions committees. All of us have written recommendation letters, given lectures in medical schools or supervised medical trainees, and counseled pre-medical students. All of us have researched medical education, albeit from different disciplinary perspectives. Drawing together these diverse perspectives, we describe some of the harmful unintended social consequences of the application process, while recognizing that readers will likely identify additional aspects of admissions’ construction ecology that deserve attention.

We proceed through discussion of three pathways that North American pre-medical applicants often follow in their quests for experiences that distinguish them as worthy applicants: following the construction-ecology metaphor, we can think of these as three supply chains for the procurement of experiences. One chain has been well developed for over a century (shadowing); one has grown remarkably in the last two decades (global-health experiences); and one is new and may turn out to be short-lived (medical scribe work). Collectively, the experiences pursued by students seeking to craft a compelling application demonstrate how the demands of admissions ramify far beyond medical schools themselves. These activities perpetuate or even exacerbate social inequalities: by laundering social capital into individual “merit” and excluding valuable applicants from the pool; by reinforcing colonizing narratives and causing harm far away; and by exploiting an already vulnerable labor force.

## Shadowing

Sources of advice directed at pre-medical students typically recommend that applicants follow or “shadow” physicians through clinical settings, an activity distinct from volunteering or paid work (Wang et al., 2015; Wilson et al., 2019; Atlantis, 2022). Today, not only medicine but virtually all health occupations expect applicants to have done some shadowing (Eades et al., 2005; Mafinejad et al., 2022; Nishi et al., 2022). The expectation that applicants demonstrate “authentic” and “informed” commitments to becoming doctors through time spent shadowing reflects medicine’s long-standing apprentice-based educational model (Aryasomayjaula et al. 2019). Pre-medical shadowing is intended to signal a commitment to undertake the rigors of physician preparation, provide an occupational “test drive” (University of Wisconsin-Madison 2022), and familiarize the applicant with the practice of health care and the physician’s role.

Critics question whether shadowing—which often amounts to a hit-or-miss 50, 20, or even just 10 hours of observation—actually does what it has been touted to do. What pre-medical students engaged in shadowing are permitted or invited to see, and what understandings they draw from such encounters, are open to question (Kitsis, 2011; Teitz, 2011). Inconsistent policies about patient privacy and safety, alongside idiosyncratic expectations of individual physicians, ensure that shadowing experiences are highly variable. Furthermore, shadowing opportunities often lack formally structured opportunities to reflect upon and make sense of one’s experiences or observations (Clark et al. 2017, Wilson et al., 2019). While studies of shadowing do exist, including scoping reviews (Kitsis & Goldsammler 2013, Thang et al., 2019), many questions remain about its impact on patient care and safety, its weighting during admission decisions, and its predictive validity.

We do not address these questions here. Rather, we focus our attention on how the demand for shadowing makes privilege appear meritorious. Whatever else shadowing does or does not accomplish, it further advantages applicants who are already privileged in terms of social class and familial connections, while discouraging promising students who lack such connections. Many institutions of medical and health professions education strongly recommend or require shadowing, but very few offer formalized pathways for finding placements. Aspiring applicants who do not already have personal and familial connections to physicians are left at a deep disadvantage, described in a 2007 study of “medical ambition” among students from underprivileged backgrounds in the UK:

Some had tried to organize their own work experience by telephoning their local hospital, but had been quickly put off by bureaucratic hurdles (e.g., being asked to call back in a week) and had not persevered. Students’ unwillingness or inability to organize work experience contrasted markedly with middle class students of similar age, who … draw extensively on the social networks of their parents to fix up informal attachments to a range of professions. (Robb et al. 2007, p. 749)

Only “two or three” of the 45 disadvantaged but high-performing students these researchers interviewed managed to arrange a shadowing opportunity.

As the generic category “shadowing” has become ever more internally stratified, different types of shadowing experiences have come to function as hierarchical markers of applicants’ initiative, industry and resourcefulness. In the endless quest to stand out, the who, what, where and how-long of shadowing has become yet one more object of applicant strategizing. Time spent with a high-profile researcher (Nobel Prize laureate or RO1 funded investigator), clinician “star” (departmental chair,school alumnus, or MD-PhD), or specialist (or sub-specialist) serves to buff one’s application to an ever-higher sheen in the quest to dazzle admissions committee members and influence their ranking decisions. In effect, shadowing can serve to launder social privilege into individual merit.

Kiel Moe describes “iatrogenic architecture” that characterizes dazzling high-rises with double glass-skin building envelopes as more “sustainable” than breathable wooden buildings, disregarding the energy consumption entailed in material manufacture, maintenance, and demolition—and the positive feedback loops involved in wood forestry and carbon sequestration (Moe, 2015). From a construction-ecology perspective, we can see that the emphasis placed on shadowing may be a form of iatrogenic architecture. It overvalues the shiny, while excluding many valuable would-be doctors who never make it to the applicant pool at all. This waste of human potential diminishes the positive feedback loops possible when members of less privileged communities become doctors.

## Global health experiences

Undergraduate “global-health experiences” (GHEs) have increased dramatically in recent decades (Merson, 2009). Universities market them (Crane, 2010) and private for-profit agencies sell them (Sullivan, 2018). Many pre-medical students believe that global-health experiences will demonstrate their altruistic service orientation, giving them an edge in admissions. GHEs are so popular that one author characterized his fellow Canadian pre-meds as “the voluntariat” (Qaiser et al., 2016). At a conference where we presented an early version of this paper, an undergraduate wept because she could not afford a volunteer opportunity abroad, and feared that her medical-school application was therefore doomed. Another claimed that “you can’t get into medical school now without deworming Somali orphans.” Some placement organizations encourage such rumors: they claim that GHEs help you “stand out from the crowd,” “be more than just an onlooker,” and gain “credible experience” for interviews (Gap Medics, 2019; Global Health PreMeds, 2014). For undergraduates, global-health experiences offer adventure, challenge—and building materials for the applications intended to position them for medical school.

Many of the undergraduate pre-health-professions students we teach fully recognize that they and their peers approach GHEs already knowing the kinds of experiences they will have, the kinds of photos they will post for friends and family, and the kinds of stories they will tell in their applications. Many seem to feel both cynical about these scripts and trapped by the perceived necessity of following them. “We call it poverty porn,” explained one. Such comments reflect a queasy awareness of the role that global-health experiences play in the construction ecology of admissions: they offer applicants access to the raw materials they believe are needed to gain admissions. The story that an applicant brings back–of a sick African orphan, or a Himalayan clinic lit by a kerosene lamp–becomes a building block for the application’s construction of a compassionate, experienced, and exceptionally committed future doctor.

The global-health experiences that provide applicants access to these raw materials have structural preconditions and consequences. Host communities can suffer both symbolic and medical harm. Linguistically incompetent and medically unprepared students—eager to be more than onlookers—may act based on the false assumption that some care is better than none (Shah & Wu, 2008; Sullivan, 2018; cf. Stone et al. 2016). Volunteers often discount the expertise or appropriate the time of foreign medical professionals (Sullivan, 2018). Students and those they encounter may be slotted into rescuer and victim roles, across economic divides that are often racialized in ways that keep old colonial tropes alive, as Naidu (2021a) points out. Global-health experiences contribute to the “white savior industrial complex” (Cole, 2012) in which other people’s complexities are flattened and international entanglements dehistoricized. The flow of students rarely runs in the opposite direction: one of us works at a school that sends many students abroad annually on global health experiences while allowing no foreign students to observe in its own hospitals and clinics, a situation that is inequitable but not uncommon. Northern students have far greater access to electives that help them “polish their curricula vitae” through “opportunities to gawk at and comment from a largely uninformed and decontextualized perspective on health care in the Global South” (Naidu, 2021a: 741).

The value of undergraduate global-health experiences for future physicians, like the value of shadowing, is not clear. Here, we want to draw attention to unintended consequences of students’ drive to demonstrate their worthiness for a demanding and selective profession through GHEs. From a construction-ecology perspective, the manufacture of global-health experiences is perhaps comparable to the harvesting of exotic hardwoods: while it can be done responsibly, it mostly comes at high environmental and social cost. One effect: the rise of the global-health experience has created an industry that may cause real harms to the already vulnerable. A second effect: given their financial and opportunity costs, GHEs reward privilege and—like shadowing—convert capital into apparent merit. A third effect: some new medical students arrive having crafted a heroic narrative they know to be disingenuous, while others may actually see themselves as heroes. All of these effects are more likely to exacerbate than to redress inequalities.

## Medical scribe work

Students who do not have the connections needed to arrange shadowing, nor the means to purchase global-health experiences, may instead spend six months to a year or more working as medical scribes in their quest to acquire experiences that they believe will help them gain admission to medical school (Abdulahad et al., 2020). Medical scribes are hired to accompany physicians in clinical settings, documenting patient encounters in real time on a tablet or laptop computer (Campbell et al., 2012). They are members of a new ‘paramedical paraprofession’ (Freidson, 1983) that has arisen to take on the considerable clerical labor created by the adoption of Electronic Health Records (EHRs) (Gellert et al., 2015; Knight, 2019; Schiff, 2016). Employing a scribe who can take on such labor is promoted as a means of preventing physician burnout, while also freeing clinicians to work directly with patients, and boosting revenue by allowing greater patient “throughput.” As one major company offering scribe services says, “Doctors save patients, and scribes save doctors” (ScribeAmerica, 2021).

The same clerical labor that is offloaded to “save” physicians, however, is presented to potential scribes as a valuable way to “jumpstart a career in healthcare” (ScribeAmerica, 2021). As one scribe company explains to prospective employees, “the wages for the position are not such that would constitute a viable long-term career option,” but “work as a medical scribe is intended to be transient … a means of gaining unrivaled clinical experience to help bridge the divide between academics and future careers in medicine” (Medical Scribes of Canada, 2021). Many sources, including the AAMC, advise would-be physicians that scribe work “will build connections with mentors who will offer you career advice, wisdom, and often, a powerful and positive letter of recommendation for your application to health professional school” (AAMC, 2021). Authors of one recent article report that many of the scribes they have worked with in their Emergency Department are in the process of applying to medical school, and “often state that shadowing experiences, while important to application committees, are increasingly difficult to arrange” (Waller, 2021). The need to acquire clinical experiences that can be reported and described in medical school applications, combined with a lack of the personal connections needed to arrange for shadowing opportunities, leads such students to compete fiercely for these otherwise unpromising jobs (Bailey, 2016).

This observation raises the uncomfortable prospect that medical school admissions may contribute to exploitative labor conditions. True, interns and residents also work hard for relatively low pay in order to advance their careers in medicine. Unlike scribes, however, residents and interns are already-admitted members of the profession who in time can expect move up in its hierarchy. Medical scribes, by contrast, undertake low-wage labor for months or years in the mere hope of someday gaining entrance into the medical profession. A recent study concluded that “many aspiring healthcare providers become medical scribes during or immediately after college, believing it will provide them with helpful experience and increase their chances of gaining entrance into medical education. However, little data exists to justify those beliefs” (Waller et al., 2021).

For those who do gain admission, the value of scribing experience for their subsequent medical training is unclear. One small qualitative study of current medical students who had previously worked as scribes found that they felt that this experience had been beneficial, confirming their commitment to medicine and teaching them some essential clinical skills. Students in their first two years, especially, expressed “higher confidence in their clinical note writing and history taking than non-scribes” (Waller et al., 2021). This confidence suggests to us that former scribes begin medical training more fully socialized into medicine and its narrative conventions; whether that is ultimately a good or bad thing may be open to discussion. Scribe work does not appear to yield any benefits in terms of medical-school class rank, USMLE (United States Medical Licensing Examination) Step 1 scores, “assessment [by others] of note writing ability, or self-reported wellbeing” (Hewlett et al., 2020).

Medical scribe work has offered a pathway toward gaining clinical experience that has been more accessible to, while arguably also exploitative of, those would-be medical school applicants who do not command the forms of social and economic capital required for physician shadowing or global-health electives. The hope of entering medical school, it seems, fills the ranks of low-paid clerical workers in medical institutions, much as the hope of making it onto a professional football team serves to fill the ranks of unpaid student-athletes on U.S. college teams.

Low-skilled laborers in construction often travel to places where incomes are higher, hoping to bring themselves and their families out of poverty. While some do find paths to authorized work, many become trapped in debt and vulnerable to unethical labor recruiters (Crates, 2016). From a construction-ecology perspective, this labor exploitation is understood as part of the overall social cost of a building. Medical scribe work directs students who begin from less elevated positions toward labor pools where a few will find access to the top reaches of medicine but most will simply keep the mill-wheels of medicine turning. The false advertising of this work as a way to “jumpstart” a career in medicine can be considered as a problematic social cost of admissions.

## Discussion

People who study admissions have long acknowledged the limitations of judging “non-cognitive” attributes through narrative self-reports (Salvatori, 2001; Kreiter et al. 2013; Ginsburg et al., 2004: Norman 2004; Kreiter, 2017). Critics have pointed to poor measurement properties and poor validity evidence (Hecker 2017). Some argue that medical-school applications incentivize lying (White et al., 2011, 2012; Siu & Reiter, 2009). Our analysis addresses a deeper problem: that even truthful accounts are built from experiences that do not help admissions committees select better future doctors, and do reinforce existing inequities and shore up damaging narratives (Dore et al., 2017; Wright, 2015; Appel, 2022). Michalec & Hafferty (2022) have described the U.S. pre-medical pathway as an example of discriminatory design, drawing from interviews with pre-medical students to show how much social, financial and cultural capital matter for people’s paths to medical school. Both clinical shadowing and global-health experiences convert these forms of capital into compelling narratives. Medical scribe work holds out the promise of experience–and success built upon that experience–to those with less capital, as an inducement to work that otherwise offers little pay or prospect for advancement.

Our point is simple. Pre-medical hopefuls seek convincing evidence of their unique fitness for the medical profession. The search for experiences that can serve as evidence of this fitness drives them to do things, and the things they do have effects that are not always in keeping with the stated values of medicine or the goal to diversify the profession. Those effects are geographically and socially far-reaching, moving beyond medical schools into would-be applicants’ families and communities, and even into distant countries. They are also fundamentally shaped by the unequal starting points from which applicants begin their journeys toward the applicant pool. Effects on would-be physicians are also profound, as they spend enormous amounts of effort, time, and money trying to meet what they believe are the entry requirements of a profession that will ultimately exclude the great majority of them, whether at the “sluice gate” of the application committee review or long before that point. Taken as a whole, these effects amount to a high human cost for a set of practices without clear value for either admissions committees or the ultimate beneficiaries—patients.

Rhetoricians who examined medical-school application essays concluded that a successful essay “reinforces the legitimacy of certain practices (humanitarian, scientific) and values (reflection and introspection), both of which become capital in the symbolic marketplace of medicine” (Bekins et al., 2004:68–69; see also Ding, 2007). In this moment, however, the legitimacy of some practices may be newly open to question. Since 2020, a devastating global virus, public battles against authoritarianism, and protests over profound racialized inequities have together cracked open the standard ways of doing business. Some for-profit global-health programs have folded under pandemic travel bans. Meanwhile, students have become attentive to calls to decolonize global health (Naidu, 2021b). Automated and offshored work has affected scribing programs; scribes now zoom in to U.S. clinical settings from India and elsewhere. Google and other companies, meanwhile, are developing artificial intelligence tools to automate scribe work, leading some to suggest that “the next step in the trend could be no human scribes at all” (Kwon, 2020). Some shadowing opportunities were derailed by COVID-19; others went virtual. Fewer students can access the spaces that once allowed applicants to build narratives about committed and altruistic selves.

In the turbulent wake and ongoing surges of these forces, some of the forms of activity that would-be physicians have mined for application essays may no longer be accessible, and others may come to be more widely seen as problematic. But the topography of the social landscape through which people get channeled toward the applicant pool remains highly uneven. From their various starting points, guided by official and unofficial advice, and by an industry eager to capitalize on consumers seeking any sliver of advantage, medical-school applicants will continue to endeavor to provide what they believe medical educators really want. If nothing changes, we can expect that privilege will still pave the path to spectacular stories; that other people’s pain will remain building material for personal ambition; and that the broader construction ecology of medical-school admissions will continue to exploit the already vulnerable and reward the already privileged.

In advocating a construction-ecology approach to building design, architect Kiel Moe notes that “the production and application of materials alter unseen ecologies, sway local and distant economies, amplify or inhibit social progress…. Only architects with an operational sense of the history, processes, and distribution of materials will sufficiently comprehend and thus alter material usage toward sustainable ends” (Moe, 2007:26). We could say the same of admissions committees: only a clear sense of the history, processes, and impacts of the various experiences would-be doctors are expected to bring to their applications will alter admissions practices to make a better profession.

Doing better will require reimagining the work of admissions. It is possible, for instance, that medical schools should consider de-emphasizing shadowing, discouraging undergraduate global-health experiences, and disputing the dubious advertising of medical-scribe companies. Going further, admissions committees may need to engage in a sustained way with the communities that the medical profession seeks to serve, to craft admissions policies that will serve the goal of inclusion as opposed to resulting in inadvertent exclusion (Razack et al., 2015) More than simply technicians manning the sluice gates, admissions committees are actors in the construction ecology of admissions. A broader view of admissions is necessary, in order to redesign it to better reflect and advance the values of the medical profession, including goals relating to equity, diversity, and inclusion. To support this redesign, admissions research must continue to expand its focus from questions of measurement and validity to an analysis of the upstream social consequences of admissions policies. The time to do better is now.
